# Photobiomodulation Regulation as One Promising Therapeutic Approach for Myocardial Infarction

**DOI:** 10.1155/2021/9962922

**Published:** 2021-07-19

**Authors:** Xinlu Gao, Wenwen Zhang, Fan Yang, Wenya Ma, Benzhi Cai

**Affiliations:** Department of Pharmacology at College of Pharmacy (State-Province Key Laboratories of Biomedicine-Pharmaceutics of China, Key Laboratory of Cardiovascular Medicine Research, Ministry of Education), and Department of Pharmacy at the Second Affiliated Hospital, Harbin Medical University, Harbin 150086, China

## Abstract

Myocardial infarction refers to myocardial necrosis caused by acute or persistent coronary ischemia and hypoxia. It is considered to be one of the significant crises threatening human health in the world. Following myocardial infarction, collagen gradually replaces the original tissue due to the loss of many cardiomyocytes, myocardial contractile function decreases, and myocardial fibrosis eventually leads to heart failure. Phototherapy is a new treatment which has shown superior efficacy on the nerve, skeletal muscle, skin, and other tissues. Likewise, there is growing evidence that phototherapy also has many positive effects on the heart. Therefore, this article introduces the progress of research on phototherapy as a new therapeutic strategy in the treatment of myocardial infarction. The wavelength of photobiomodulation in the treatment of myocardial infarction is specific, and the influence of light source power and light duration on the tissue presents a bell-shaped distribution. Under these conditions, phototherapy can promote ATP synthesis and angiogenesis, inhibit the inflammatory response, improve heart function, reduce infarct size, and protect myocardium. In addition, we summarized the molecular mechanisms of phototherapy. According to the location of photoreceptors, they can be divided into mitochondrial and nonmitochondrial parts.

## 1. Introduction

Myocardial infarction (MI) is defined as myocardial necrosis caused by acute or persistent coronary ischemia and hypoxia. It is considered one of the major crises to human health worldwide [1, 2]. MI will be accompanied by many adverse events. Typically, the heart spontaneously remodels and leads to hypertrophy in response to a lack of systemic blood supply. The severity of MI increases, eventually leading to heart failure and life crisis [3, 4]. However, myocardial remodeling after MI is a complicated process accompanied by an inflammatory phase which usually occurs 1-3 days after MI [5]. Numerous inflammatory cytokines are recruited to the infarct site, leading to immune cell infiltration and elimination of irreversible tissue damage. As the inflammation subsides, myocardial remodeling enters the repair phase, during which myofibroblast proliferation, extracellular matrix deposition, angiogenesis, and scar repair occur [6]. However, it is generally difficult to achieve a balance between inflammation and repair, such as excessive delays in the inflammatory phase or early remission of the repair phase, which will lead to aggravated myocardial injury, often characterized by left ventricular distention, ventricular wall thinning, myocardial fibrosis, myocardial dysfunction, tissue inflammation, oxidative stress, apoptosis, calcium overload, and abnormal energy metabolism [7]. In addition, the complete cure rate of MI is minimal, and the recovery is poor. Many patients suffer from MI accompanied by neurological damage, leading to cognitive dysfunction [8]. Recently, many new technologies have been developed for the treatment of MI. These treatments have been progressively upgraded from basic coronary event monitoring to aggressive prevention and treatment of potential coronary artery thrombosis [9]. According to a rising number of studies, targeted drugs, exercise training, cell therapy, and various interventional techniques can reduce the size of myocardial fibrosis and improve cardiac function in experimental animals and clinical humans with MI. However, potential adverse reactions or difficulties in clinical conversion remain unresolved bottlenecks of these MI treatment strategies. Therefore, a noninvasive, nonpharmacological, and clinically applicable technical approach to improve myocardial ischemia and ensure the survival rate of patients with MI is urgently needed.

In recent years, experimental studies have shown that photobiomodulation (PBM) is a promising approach that can regulate various biological processes [10]. PBM refers to low-power (1-500 mW) nonthermally transferred photons with a spectrum in the visible light or near-infrared (400-1000 nm) range that can trigger beneficial biological reactions in cells and tissues [11]. The term “photobiomodulation” has been refined through decades of continuous research from the initial “photobiological activation” to “biological stimulation,” and then to the “biological regulation” in which light has a bidirectional regulatory effect on biological organisms [12, 13]. Until recently, PBM was collectively referred to as the term of light regulating biological organisms [14]. The bidirectional modulatory PBM that activates or inhibits biological organisms includes the initial dominant low-level laser therapy (LLLT) and light-emitting diode therapy (LEDT) with more performance advantages. PBM primarily uses low-power light beams at specific wavelengths to stimulate biological organisms to produce physical responses [15, 16]. It generally restores the body's pathological state to normal and achieves the treatment of diseases by adjusting the body's immune system, nervous system, blood circulation system, and multiple tissue metabolism systems [17–19]. Currently, PBM is used clinically in the treatment of various skin diseases such as psoriasis, eczema, and leucoderma. At the same time, it has also played a beneficial role in promoting wound healing in neonatal jaundice, joint inflammation, and fracture healing [20–24]. Various animal experiments have also demonstrated that Alzheimer's disease, fatigue, and pain have improved after being treated by PBM, such as satellite cells in skeletal muscle tissue, and neural stem cell-related cell proliferation factors can be activated by LLLT [25, 26]. Red and near infrared light at 630-650 nm is the main light source used in PBM [27]. Earlier, Horvath et al. used a laser to reduce the size of MI by a novel method of making miniature cells in the sheep's myocardium [28]. More importantly, a growing body of experimental evidence suggests that the application of PBM combined with conventional cardiac interventions is more efficient when it comes to repairing and improving MI-related disease [29]. Therefore, it is necessary to specifically discuss the application of PBM in myocardial infarction and summarize the underlying mechanisms and metabolic signaling pathways in PBM. The purpose of this review is to systematically summarize previous experimental evidence on the use of PBM for MI in tissue studies and animal experiments.

This provides researchers and clinicians with potential theories for further experimental research, as well as a theoretical basis for the clinical application and promotion of PBM.

## 2. Materials and Methods

### 2.1. Search Strategy

The published articles related to photobiomodulation and heart disease were searched in PubMed, Web of Science, and Google Scholar databases. The keywords included “photobiomodulation,” “phototherapy,” “low-level laser therapy,” “laser therapy,” “low-level laser radiation,” “light emitting diode,” “myocardial infarction,” and “heart failure,” and the MeSH terms were used individually or in combination with increasing the findings. The language of the article is limited to English.

### 2.2. Outcome Selection

Studies include the following: (1) light source equipment, light parameters (wavelength, power density, energy density, and light time), and irradiation mode; (2) experimental animals; (3) improved phenotypes of myocardial diseases, such as infarct size, myocardial contractile function, and inflammatory response; and (4), in addition, the cellular and molecular effects of photobiological regulation which are also results that are of interest to us.

## 3. Results

### 3.1. PBM-Related Parameters

#### 3.1.1. Light Sources

The light source equipment of PBM in MI is classified as coherent laser and incoherent semiconductor light-emitting diodes (LED). According to experimental designs, different light sources are applied. After the first laser was discovered, people started to adopt them in medical applications [30]. As early as 1988, researchers confirmed the positive effect of low-energy laser on the primary pathogenesis of acute myocardial infarction. It mainly relieves pain, stabilizes the cardiac electrophysiology, reduces the area of myocardial ischemia injury, and thereby promotes scar formation [31]. Subsequently, LLLT has been continuously applied to MI experimental research. Yaakobi et al. used Ga-As lasers with a wavelength of 805 ± 5 nm, and Bibikova et al. used He-Ne lasers with a wavelength of 630 ± 5 nm both proved that PBM can adjust the myocardial remodeling reaction process after MI [32]. In addition, with the advent of LEDs, several studies have demonstrated that 630-660 nm LEDs have the same effect as lasers in the treatment of various diseases [15, 33, 34]. It has been confirmed that the working mechanism of PBM is independent of the thermal effect of the light source [24]. Therefore, there is no essential difference between coherent lasers and incoherent LEDs in the applications of light sources. The development of light stimulation on repairing myocardial infarction injury (MIR) depends on many factors, such as power density, frequency, and duration of laser irradiation of biological tissue. According to previous reports, the main reason for using radiation in the red and near-infrared spectral region is that hemoglobin is not absorbed in this region, and red light is more likely to exert biological effects through tissues [35]. Related studies have reported that light radiation has a biphasic modulatory effect on the myocardium, with positive impacts within a certain power range, beyond which no practical influence can be exerted [36, 37]. Mousquès and Chairay found that the “optimal” range for laser on the ischemic heart is 5-15 mW/cm^2^; the laser-induced angiogenesis effect is also the best in this power range [38]. This is consistent with studies regarding the power range of laser suppression of infarct size [39]. Furthermore, another research showed that 25 mW/cm^2^ laser had the minimal effect on reducing infarct size [40]. However, the biphasic adjustment effect of PBM on the body has been verified in another way. Bibikova et al. stated that too much frequency of laser irradiation at a specific power density is not beneficial to body tissues. Multiple irradiations of skeletal muscle cells, either daily or every other day irradiation, at doses that did not show obvious proliferation-promoting effect [19]. The biological body's exposure time is also limited, and power density and time have a bell-shaped distribution on the body's efficacy. The short duration of irradiation can exert beneficial biological effects, but excessively long time exposure is detrimental, which may be related to the fact that light stimulates the body to produce excessive reactive oxygen species (ROS). All these studies proved that comprehensive consideration of the light dose and light duration used in the treatment method will have great significance on the therapeutic effect.

#### 3.1.2. Experimental Animals and Irradiation Methods

Stage studies of PBM repair of MIR are mainly based on animal experimental models. Experimental animals have been reported in toads, rats, mice, rabbits, pigs, and dogs, mainly in rats [41–43]. All animal experiments have shown the excellent effects of PBM. The light was used to irradiate the infarcted myocardium immediately after MI, and then the myocardial tissue was transcutaneously irradiated again after a few days [37]. In addition, Biasibetti et al. suggested that the heart was indirectly regulated by irradiating the gastrocnemius muscle of the rat's leg. And Tuby et al. found that the infarct size could be reduced by injecting red light-stimulated autologous mesenchymal stem cells into the infarcted myocardium [44, 45].

### 3.2. Application of PBM in Myocardial Infarction

#### 3.2.1. PBM Improves Myocardial Structure after MI

The purpose of ventricular remodeling after MI is to adapt to the adverse effects of myocardial ischemia, leading to concentric hypertrophy. However, this stress response is transient and will eventually develop into heart failure as the heart function continues to deteriorate. Reducing the infarct size is a beneficial way to relieve cardiac dilation, improve ventricular function, and delay the development of heart failure. In this regard, many studies have analyzed the important role of PBM after myocardial ischemia [46]. Yaakobi et al. proved the positive effect of PBM on reducing infarct size. After ligating the left anterior descending coronary artery of the animal heart and performing photostimulation, the low-power density laser can significantly reduce the infarct size. The reduction rate gradually increased with the duration of MI. In addition, the dilated volume of the left ventricle also decreased, showing a long-term effect of LLLT in repairing MI tissue [32]. Ad and Oron also observed that PBM could prolong the survival rate of MI animals, reducing the mortality to less than 6.5%, and significantly extending the time cycle of animal deaths after MI. Furthermore, the authors also reported that PBM could significantly reduce mortality, hinder the progression of MI to heart failure, and improve survival [46]. At the same time, echocardiography showed an increase in both myocardial ejection fraction and shortening fraction compared to the untreated group. The left ventricular internal diameter at end-systole (LVID) and left ventricular internal diameter at end-systole (LVIDd) have also been shown to be adjusted down in some studies. However, it has also been noted that PBM has little effect on the mortality of MI. The researcher believed that the previous research results may have been influenced by the heterogeneity of MI models [45, 47].

#### 3.2.2. PBM Reduces Mitochondrial Damage after MI

Mitochondria are a key factor in regulating the health of cardiomyocytes and tissues and occupy a central position in the function of myocardial tissue. The heart consumes large amounts of adenosine triphosphate (ATP) during normal diastolic and systolic, and it is the most ATP-consuming organ other than the skeletal muscle. When the myocardium is damaged, the structure of myocardial mitochondria changes, with swollen mitochondria and reduced mitochondrial cristae, contributing to the reduction in ATP synthesis. In Oron's study, mitochondrial morphology and ATP production in myocardial tissue were measured after light exposure. It was founded that PBM could reduce mitochondrial damage by 60%, including mitochondrial swelling, vacuolization, and mitochondrial spinal injury [47]. This is consistent with the observation of increased ATP production in the ischemic myocardium, demonstrating that PBM can inhibit the rapid decline of ATP and reverse the results of insufficient myocardial energy [32].

#### 3.2.3. PBM Regulates the Expression of Inflammatory Factors after MI

After an acute MI, many proinflammatory factors accumulate in the injury zone so that the imbalance between pro- and anti-inflammatory cytokines triggers a series of inflammatory cascades. Hentschke's team found that PBM could stimulate the gastrocnemius muscle of MI rat with 3 J/cm^2^ light for 10 consecutive days without significant regulation of Interleukin-6 (IL-6) and Interleukin-10 (IL-10) in rat serum. However, at an energy density of 21 J/cm^2^, IL-6 and tumour necrosis factor-alpha (TNF-*α*) decreased. Simultaneously, IL-10 increased significantly, so the ratio of IL-6/IL-10 decreased correspondingly [43]. Manchini et al. also proved that a light source of 22.5 J/cm^2^ could directly illuminate the MI tissue and inhibit IL-1 and IL-6 mRNA [39]. The reduction of the inflammatory response can weaken the infiltration of white blood cells in the infarct area and reduce the adverse effects produced in this process [48].

#### 3.2.4. PBM Promotes Angiogenesis after MI

PBM can not only directly exert the positive effects of MIR treatment on myocardial repair, ATP synthesis, and regulation of inflammatory factors but also facilitates myocardial protection [49]. PBM has been reported to regulate vascular endothelial growth factor (VEGF), and nitric oxide synthase (NOS) synthesis can stimulate endothelial cell proliferation and promote angiogenesis within the infarct broad zone [32]. Angiogenesis can deliver oxygen and nutrients to myocardial tissue and play a role in metabolic processes [32, 38].

### 3.3. Molecular Mechanism of PBM in MI Treatment

**Figure 1:**
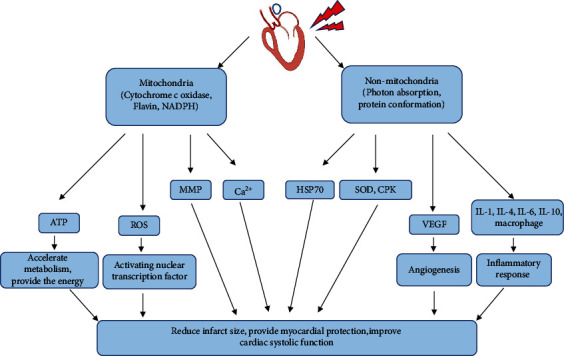
Schematic diagram of myocardial tissue regulated by photobiomodulation.

At present, the researches on PBM for MIR are mainly aimed at improving cardiac function and phenotype, and the more profound mechanism research remains to be explored. This review has summarized the molecular mechanisms involved in the research of experimental studies of PBM for MI into the following several aspects (Figure 1).

First, Lin et al. demonstrated that the effect of LLLT therapy was not induced by thermal effects, but by as yet an undefined process known as “photobiological stimulation” [50]. Tali et al. inserted probes into the irradiated myocardium, and there was no temperature rise in the rat myocardium irradiated with laser for 3 min. Meitian et al. detected the temperature of the culture medium under the light. Compared with the dark control, the local temperature rise was less than 0.1°C and almost negligible, which eliminated the thermal effect of light [24].

#### 3.3.1. Mitochondria

Typically, the light only works after it is absorbed by the chromophores of endogenous cells or tissues. As the functional center of the cell, mitochondria can converge various signals between organelles and the nucleus. As early as 1981, it was suggested that photosensitivity might be a common mitochondrial characteristic in higher animals [37]. The general trend of many studies points to the terminal enzyme of the mitochondrial electron transport chain-cytochrome C oxidase, which may be the receptor for mitochondria to receive light stimulation signals, and has a key role in the treatment of MIR by PBM. In other cell types, red visible and near-infrared light irradiations have also been found to activate components of the mitochondrial respiratory chain, initiating signaling cascades that promote cell proliferation and cytoprotection [51–53]. Cytochrome C oxidase accelerates the transmission of electrons in the electronic respiratory chain after receiving light signals and increases the mitochondrial membrane potential and thus promotes ATP synthesis [54]. In addition, after mitochondria receive light signals, they first cause a change in the configuration of the photoreceptor molecule and then triggers a secondary reaction-activation of mitochondrial retrograde signals [55]. These cellular and biological changes give rise to macroscopic effects, such as repair damage and promote proliferation [56].

#### 3.3.2. Nonmitochondrial

PBM plays a regulatory effect on a variety of nonmitochondrial signaling pathways that are associated with reducing adverse cellular stress responses, improving tissue structure, and restoring homeostasis. The nonmitochondrial effects of PBM involve signal pathways originating from the absorption of photons on the cell membrane and the regulation of protein conformation. Various photoactive molecules can absorb photons in ion channels and membrane receptors. Downstream signal transduction affects inflammatory cytokines, oxidative stress, growth factors, and cytoprotective factors [57].

The balance between pro- and anti-inflammatory cytokines is critical to the prognosis after MIR, as the inflammatory response is the prerequisite basis for wound healing and scar formation. Excessive inflammation can cause secondary damage to the myocardium, but PBM appears to affect the level of inflammation, thereby acting cardioprotective effect. IL-1, IL-4, IL-6, IL-10, and macrophages are all involved in the inflammatory response after MI [39]. The beneficial regulation of PBM on oxidative stress may be achieved by the heat shock protein (HSP70). Yaakobi et al. reported that LLLT increased myocardial HSP70 by 2.2-fold in the rat ischemia-reperfusion model [32]. Aboujaoude et al. reported that superoxide dismutase (SOD) and creatine phosphokinase (CPK) increased sharply in a short period after PBM stimulation and suggested that this increase was beneficial to the repair of MI [58]. Many studies have found that PBM promotes angiogenesis in the boarding zone of infarction and is accompanied by an increase in vascular endothelial growth factor (VEGF). In addition, nitric oxide (NO) and inducible nitric oxide synthase (iNOS) are also involved in cardiac injury. Tuby et al. reported that iNOS increased significantly with the increasing dose of PBM and reached a peak at 2 days, indicating that NO continues to play a role after acute injury [59].

## 4. Discussion

The study of PBM treatment of MI is a multidisciplinary and multilevel intersection that includes comprehensive experimental research on optics, mechanics, physiology, and biochemistry. The actual operation is influenced by the light source equipment, lighting parameters (wavelength, power, frequency, and spot area), irradiation location, and treatment modalities. The experimental factors need to be considered from multiple aspects. Initially, laser radiation was mostly used for PBM therapy. The advantages of using a laser beam as the treatment light source are high coherence, monochromatic wavelength and stability, and strong penetration ability to tissue. However, the laser is also characterized by heavy equipment, high cost, and low safety, and in practical applications, they can also produce certain specific thermal effects to interfere with the experimental results. LED light sources have advantages in materials such as low cost, good portability, ease of use, low energy consumption, and long life of diode light sources. Moreover, compared with lasers, LEDs have a wider radiation range and higher safety and have gradually developed from the lighting equipment used at home to the medical field. The wider bandwidth and more variable wavelengths, amplitudes, and phases allow the energy absorbed by each cell tissue irradiated by LEDs to remain uniform, and almost no heat is generated. It is worth mentioning that LEDs have been shown to be as effective as lasers in bioregulation [60]. Therefore, both laser light sources and LEDs are currently being used in experiments. Red-light phototherapy with its high wavelength selectivity, high controllability, and no heat energy generation deserves further exploration in future medical treatment.

Most PBM researches have been limited to the level of animal experiments. In the transition to clinical, due to the differences in the physiological structure of animal models such as humans, mice, and dogs, the energy penetration and absorption of light sources on animal and human body tissues and dosage selection need to be considered. Gepstein et al. and Kornowski et al. proposed to transmit the optical fiber in catheters to the human myocardium via a nonfluoroscopic intracorporeal navigation and positioning technology and designed the technology of optical fiber transmission for laser transmission and percutaneous puncture for the pretreatment of the LLLT myocardial surface [61, 62]. In addition, the noninvasive transdermal illumination methods are also promising for research, which can reduce damage to the human body and are easy to operate.

Moreover, there are still many problems that need to be clarified regarding the mechanism of PBM in the treatment of myocardial injury. As the research of PBM in the cardiac field continues to increase, many articles have been published in this field, but there may be some differences in these results. These discrepancies may be due to differences in research design, including different light sources and parameters inequality of choice. Therefore, we need to review multiple studies of photodynamic therapy in the future, summarize the results of previous PBM treatment for ischemic cardiomyopathy, analyze the differences between individual experimental studies, provide reliable experimental guidance for future PBM treatment, and establish standardized clinical parameters as a valid reference.

## 5. Conclusion

This review systematically summarizes the role of PBM in cardiac injury and elaborates on the selection of light sources, experimental animals, light treatment methods, therapeutic indicators, and potential molecular mechanisms. These summaries provide systematic reference suggestions for future light modulation of myocardial physiological functions. In summary, PBM can repair MIR by improving cardiac function, reducing the size of myocardial fibrosis, promoting vascular regeneration of the injured myocardial area, and regulating inflammatory factors and certain protective factors. Compared with other treatment options for MI, PBM has the advantages of noninvasive, nonpharmacological, nonhereditary, and low difficulty in applying technology and has excellent potential in cardiac therapy.
